# Health and Rest

**Published:** 1900-09

**Authors:** 


### Health and Rest.

Among the many institutions whose object is perfect rest and physical and mental recuperation, none ranks more highly than the Alma Sanitarium, Alma, Michigan. Situated in a region unsurpassed in healthful conditions, the establishment offers every conceivable facility in the way of superior equipment, sources of recreation, and skillful and assistant treatment.

A leading feature of the institution,—which of itself would guarantee perfect recovery in many cases of invalidism—is its inexhaustible supply of Nature's remedy, known as Alma Bromo Water. This remarkable bromide possesses properties unshared by other similar productions of nature, being a peculiarly efficient laxative, and, as an ingredient of the bath, wonderfully effectual in the treatment of chronic rheumatism, while as an excellent sedative in nervous and skin diseases, experience has proved it to be absolutely without an equal.

The Park Mineral Spring is highly efficacious, its exhilarating effects being such that it is a popular beverage among guests, who imperceptibly find their mental and bodily powers renewed under its peculiar influence. Even those not requiring medical treatment, but who resort to the Sanitarium to enjoy its provisions for freedom from care and ideal rest, find themselves notably reinvigorated by the subtle yet marvelously potent effects of this unique diuretic water.

Aside from the signal advantages of the waters above mentioned, the Sanitarium offers the rarest opportunities for healthful recreation and pleasure. The neighboring country affords charming attractions in the way of walks and drives; amusements of every description have been liberally planned, and the general atmosphere of the place is such as to inspire cheerfulness and contentment even among those long prostrated by chronic disease.

The institution is thoroughly equipped in each department, having electricity in every form, massage, rest cure, manual and mechanical Swedish movements, and a well equipped gymnasium under a trained director.

We do not hesitate to recommend Alma Sanitarium cordially and confidently, knowing its high character and the sterling value of its manifold resources as a health resort.

